# Associations between abdominal obesity indices and diabetic complications: Chinese visceral adiposity index and neck circumference

**DOI:** 10.1186/s12933-020-01095-4

**Published:** 2020-07-31

**Authors:** Heng Wan, Yuying Wang, Qian Xiang, Sijie Fang, Yi Chen, Chi Chen, Wen Zhang, Haojie Zhang, Fangzhen Xia, Ningjian Wang, Yingli Lu

**Affiliations:** 1 Institute and Department of Endocrinology and Metabolism, Shanghai Ninth People’s Hospital, Shanghai Jiao Tong University School of Medicine, Shanghai, 200011 China; 2 Department of Endocrinology, The Fifth Affiliated Hospital of Kunming Medical University, Yunnan Honghe Prefecture Central Hospital (Ge Jiu People’s Hospital), Yunnan, China; 3 Department of Ophthalmology, Shanghai Ninth People’s Hospital, Shanghai Jiao Tong University School of Medicine, Shanghai, China

**Keywords:** Diabetic complications, Cardiovascular and cerebrovascular disease, Diabetic kidney disease, Abdominal obesity, Chinese visceral adiposity index, Neck circumference

## Abstract

**Background and aims:**

Obesity, especially abdominal obesity, has been considered a risk factor for diabetic complications. Many abdominal obesity indices have been established, including neck circumference (NC), waist-to-hip ratio (WHR), lipid accumulation product (LAP), visceral adiposity index (VAI) and the Chinese visceral adiposity index (CVAI). However, studies investigating the associations between these indices and diabetic complications are limited. The objective of this study was to investigate the associations of the abdominal obesity indices with cardiovascular and cerebrovascular disease (CVD), diabetic kidney disease (DKD) and diabetic retinopathy (DR).

**Methods:**

A total of 4658 diabetic participants were enrolled from seven communities in Shanghai, China, in 2018. Participants completed questionnaires and underwent blood pressure, glucose, lipid profile, and urine albumin/creatinine ratio measurements; fundus photographs; and anthropometric parameters, including height, weight, waist circumference (WC), NC and hip circumference (HC).

**Results:**

In men, a one standard deviation (SD) increase in CVAI level was significantly associated with a greater prevalence of CVD (OR 1.35; 95% CI 1.13, 1.62) and DKD (OR 1.38; 95% CI 1.12, 1.70) (both *P* < 0.05). In women, a one SD increase in CVAI level was significantly associated with a greater prevalence of CVD (OR 1.32; 95% CI 1.04, 1.69) and DKD (OR 2.50; 95% CI 1.81, 3.47) (both *P* < 0.05). A one SD increase in NC was significantly associated with a greater prevalence of CCA plaque in both men (OR 1.26; 95% CI 1.10, 1.44) and women (OR 1.20; 95% CI 1.07, 1.35). These associations were all adjusted for potential confounding factors.

**Conclusions:**

CVAI was most strongly associated with the prevalence of CVD and DKD among the abdominal obesity indices, and NC was unique associated with the prevalence of CCA plaque in Chinese adults with diabetes.

*Trial registration* ChiCTR1800017573, www.chictr.org.cn. Registered 04 August 2018.

## Introduction

Epidemiological data show that the worldwide prevalence of overweight or obesity, which has reached nearly 33.3%, has doubled since 1980 [1]. Both overweight and obesity have been considered risk factors for hemodynamic, endothelial, or inflammatory disorders [2], type 2 diabetes (T2DM) and its complications [3, 4], and even all-cause mortality [5]. Interestingly, some studies have determined that the distribution of adipose tissue rather than the amount may play a more crucial role in the development of vascular complications [4–6].

In fact, the methods to detect abdominal adiposity include dual-energy X-ray absorptiometry (DEXA), computed tomography (CT), magnetic resonance imaging (MRI) and dual bioelectrical impedance analysis (BIA) [7, 8], which are unsuitable for routine clinical practices in a general population on account of the radiation exposure, time requirements and high costs [9, 10]. Thus, many indices to estimate central or abdominal obesity have been established, including the visceral adiposity index (VAI) and the lipid accumulation product (LAP), which are calculated using the data of WC, BMI, triglycerides (TG), and high-density lipoprotein (HDL) [11]. It should be noted that the Chinese visceral adiposity index (CVAI) is a recently established indicator of abdominal obesity based on age, BMI, WC, and metabolic parameters, and it has been considered to serve as a better predictor of T2DM and prediabetes than the VAI, BMI, waist circumference (WC) and/or waist to hip ratio (WHR) in Chinese adults [12, 13]. Furthermore, neck circumference (NC), a stable marker for determining upper body subcutaneous adipose tissue distribution, has also been considered an anthropometric indicator of abdominal obesity [11, 14].

T2DM is a chronic life-threatening disease, leading to macro- and microvascular complications including cardiovascular and cerebrovascular disease (CVD), diabetic kidney disease (DKD) and diabetic retinopathy (DR), which are associated with increased disability and reduced quality of life and life expectancy [15]. To our knowledge, the evidence for the associations of NC, LAP, VAI and CVAI with CVD in adult with diabetes are still limited. Furthermore, the associations of generalized obesity and abdominal obesity with DKD and DR are inconsistent. For instance, one recent study found that individuals with generalized obesity rather than specifically abdominal obesity were more likely to have DKD than underweight or normal weight individuals [16]. However, another study reported that, compared with generalized obesity, abdominal obesity was more closely associated with DKD [17]. Earlier studies suggested that higher BMI increased DR risk [18, 19], although recent studies reported either no association [20] or a negative association between BMI and the prevalence of DR [21].

Thus, in this study including a large community-based sample, we aimed to investigate the associations of abdominal obesity indices, including WC, NC, WHR, LAP, VAI and CVAI with the prevalence of CVD, DKD and DR in Chinese diabetic adults. Our findings may provide evidence for the early detection, prevention and treatment of diabetic complications.

## Materials and methods

### Study design and participants

This cross-sectional study was designed to investigate the associations between obesity phenotype indices and diabetic complications among Chinese adults. Detailed protocols have been described in previously published papers [22–24]. In brief, participants who were previously diagnosed with diabetes and had lived in their current area for ≥ 6 months were enrolled from seven communities in Huangpu and Pudong Districts, Shanghai, China. In August 2018, 4813 participants (23–99 years old) with diabetes received examinations, and participants who were missing WC, NC or HC measurements (n = 154) or missing TG or HDL data (n = 1) were excluded. In total, 4658 participants were involved in the final analyses (Additional file 1: Figure S1).

The study protocol was approved by the Ethics Committee of Shanghai Ninth People’s Hospital, Shanghai Jiao Tong University School of Medicine. All procedures were performed in accordance with the ethical guidelines of the 1975 Declaration of Helsinki and the ethical standards of the responsible institutional and national committee on human experimentation. We obtained written consent from all participants enrolled in the study.

### Data collection

The information on sociodemographic characteristics, medical history, family history, and lifestyle factors was accessed by the same group of trained and experienced personnel from the SPECT-China study [25] through a face-to-face interview using a detailed questionnaire. Individuals who have smoked at least 100 cigarettes in their lifetime and who were currently smoking cigarettes were defined as current smokers [26].

Anthropometric measurements including weight, height, NC, WC, hip circumference (HC) and blood pressure were conducted by trained staff according to standard protocols as described previously [11]. Height and weight were measured with participants standing without shoes and in lightweight clothes to the nearest 0.1 cm and 0.1 kg. WC was measured on the midaxillary line between the lowest border of the rib cage and the top of the iliac crest to the nearest 0.1 cm. NC was measured below the cricoid cartilage and then at the level of the mid-cervical spine to the nearest 0.1 cm. HC was measured at the widest part of the hip at the level of the greater trochanter to the nearest 0.1 cm. Blood pressure was measured in the nondominant arm by an automated electronic device (HEM-752 FUZZY, Omron, China). After a 5-min rest, blood pressure measurements were repeated three times with 1-min intervals. The average systolic and diastolic blood pressures of the three readings were recorded on the questionnaire. BMI was calculated as weight in kilograms divided by squared height in meters. WHR was calculated as waist circumference divided by hip circumference. The LAP, VAI and CVAI were calculated as follows:

$$ \begin{aligned} {\text{Males}}:{\text{VAI}} & = {\text{WC}}\left( {\text{cm}} \right)/\left[ { 3 9. 6 8+ 1. 8 8\times {\text{BMI}}\left( {{\text{kg/m}}^{ 2} } \right)} \right] \times \left[ {{\text{TG}}\left( {{\text{mmol}}/{\text{L}}} \right)/ 1.0 3} \right] \\ & \quad \times \left[ { 1. 3 1/{\text{HDL}}\left( {{\text{mmol}}/{\text{L}}} \right)} \right] \\ \end{aligned} $$

$$ {\text{LAP}} = \left[ {{\text{WC}}\left( {\text{cm}} \right){-} 6 5} \right] \times {\text{TG}}\left( {{\text{mmol}}/{\text{L}}} \right) $$

$$ \begin{aligned} {\text{CVAI}} & = - 2 6 7. 9 3+ 0. 6 8\times {\text{age}}\left( {\text{y}} \right) + 0.0 3\times {\text{BMI}}\left( {{\text{kg}}/{\text{m}}^{ 2} } \right) + 4.00 \times {\text{WC}}\left( {\text{cm}} \right) \\ & \quad + 2 2.00 \times {\text{Lg TG}}\left( {{\text{mmol}}/{\text{L}}} \right) - 1 6. 3 2\times {\text{HDL}}\left( {{\text{mmol}}/{\text{L}}} \right) \\ \end{aligned} $$

$$ \begin{aligned} {\text{Females}}:{\text{ VAI}} & = {\text{WC}}\left( {\text{cm}} \right)/\left[ { 3 6. 5 8+ 1. 8 9\times {\text{BMI}}\left( {{\text{kg}}/{\text{m}}^{ 2} } \right)} \right] \times \left[ {{\text{TG}}\left( {{\text{mmol}}/{\text{L}}} \right)/0. 8 1} \right] \\ & \quad \times \left[ { 1. 5 2/{\text{HDL}}\left( {{\text{mmol}}/{\text{L}}} \right)} \right] \\ \end{aligned} $$

$$ {\text{LAP}} = \left[ {{\text{WC}}\left( {\text{cm}} \right){-} 5 8} \right] \times {\text{TG}}\left( {{\text{mmol}}/{\text{L}}} \right) $$

$$ \begin{aligned} {\text{CVAI}} & = - 1 8 7. 3 2+ 1. 7 1\times {\text{age}}\left( {\text{y}} \right) + 4. 3 2\times {\text{BMI}}\left( {{\text{kg}}/{\text{m}}^{ 2} } \right) + 1. 1 2\times {\text{WC}}\left( {\text{cm}} \right) \\ & \quad + 3 9. 7 6\times {\text{Lg TG}}\left( {{\text{mmol}}/{\text{L}}} \right) - 1 1. 6 6\times {\text{HDL}}\left( {{\text{mmol}}/{\text{L}}} \right). \\ \end{aligned} $$

### Biochemical measurements

Blood samples were drawn between 6:00 am and 9:00 am after an overnight fast of at least 8 h. The blood samples for the plasma glucose test were collected into vacuum tubes with the anticoagulant sodium fluoride and centrifuged within 2 h after collection. The serum was aliquoted and frozen at − 20 °C after collection and then shipped by air within 2–4 h on dry ice to a central laboratory. Glycated hemoglobin (HbA1c) was assessed by high-performance liquid chromatography (MQ-2000PT, Medconn, China). Fasting plasma glucose (FPG), serum creatinine, triglycerides, total cholesterol, HDL and low-density lipoprotein (LDL) were performed with a Beckman Coulter AU 680 (Brea, USA).

Morning fasting spot urine samples were refrigerated immediately and frozen at − 20 °C within 2 h. The concentrations of urine albumin and creatinine were measured with a Beckman Coulter AU 680 (Brea, USA) using a turbidimetric immunoassay and an enzymatic method in a single spot urine sample, respectively, after which the urine albumin/creatinine ratio (ACR) was calculated.

DR screening was accessed by mydriatic binocular indirect ophthalmoscopy (Topcon TRC-NW400 Non-Mydriatic Retinal Camera, Oakland, USA). Fundus photographs were read by a retina specialist ophthalmologist.

### Definition of variables

Hypertension was defined as systolic blood pressure ≥ 140 mmHg, diastolic blood pressure ≥ 90 mmHg, or a self-reported previous diagnosis of hypertension. Dyslipidemia was defined as total cholesterol ≥ 6.22 mmol/L (240 mg/dL), triglycerides ≥ 2.26 mmol/L (200 mg/dL), LDL ≥ 4.14 mmol/L (160 mg/dL), HDL < 1.04 mmol/L (40 mg/dL), or a self-reported previous diagnosis of hyperlipidemia by a physician, according to the modified National Cholesterol Education Program-Adult Treatment Panel III [27].

The CVD outcome was defined as a previous diagnosis of coronary heart disease, stroke, or peripheral arterial disease and was recorded in the registration platform [28].

The estimated glomerular filtration rate (eGFR) was calculated according to the Chronic Kidney Disease Epidemiology Collaboration (CKD-EPI) equation for “Asian origin” [29]. The definition of DKD was ACR higher than 30 mg/g or eGFR < 60 mL/min per 1.73 m^2^, as suggested by the statement from the American Diabetes Association [30].

Participants without DR were defined as having no abnormalities in fundus photographs; participants with DR included individuals with intraretinal microaneurysms, hemorrhages, venous beading, prominent microvascular abnormalities, neovascularization or vitreous/preretinal hemorrhages in accordance with the Global Diabetic Retinopathy Project Group [31].

### Statistical analysis

Data analyses were performed with IBM SPSS Statistics, version 26 (IBM Corporation, Armonk, NY, USA). *P* value (two-sided) < 0.05 indicated significance. Continuous variables were expressed as the mean ± standard deviation (SD) or the median with an interquartile range (25%, 75%), and categorical variables were presented as percentages (%). The Mann–Whitney U test or Student’s t test and Chi-square test were used for continuous and dichotomous variables, respectively. In the analyses, the concentrations of urinary ACR were logarithmically transformed to achieve a normal distribution.

Regression tests were used to analyze the associations between abdominal obesity indices and diabetic complications. Data were summarized as odds ratios or regression coefficients (95% CI). The associations of WC, NC, WHR, LAP, VAI and CVAI with the prevalence of common carotid artery (CCA) plaques, CVD, DKD and DR were detected by binary logistic regression analyses. Linear regression analysis was used to detect the associations of WC, NC, WHR, LAP, VAI and CVAI with Ln ACR and eGFR. Adjustment variables were tested by collinearity diagnosis according to the following criteria: variance inflation factor (VIF) > 10 or tolerance near 0.1; condition index > 30; and variance proportions > 50%. The variables without collinearity were selected.

Finally, the associations of WC, NC, WHR, LAP, VAI and CVAI with CCA plaque, CVD, DKD, DR and Ln ACR were adjusted for adjusted for age, education, duration of diabetes, current smoking, BMI, HbA1c, LDL and systolic blood pressure. The associations of WC, NC, WHR, LAP, VAI and CVAI with eGFR were adjusted for education, duration of diabetes, current smoking, BMI, HbA1c, LDL and systolic blood pressure. Receiver operating characteristic (ROC) curve analysis was used to compare the prognostic powers of BMI, WC, NC, WHR, LAP, VAI and CVAI for CVD and DKD among men and women. The pairwise comparison of the area under the ROC curve of these abdominal obesity indices was analyzed by the Z test.

Sensitivity analyses were performed in the supplementary materials. To further analyze whether BMI had an effect on these associations, we investigated the associations between abdominal obesity indices and the prevalence of diabetic complications without adjusting for BMI. The association between BMI and the prevalence of diabetic complications was also investigated (Additional file 2: Table S1). Then, abdominal obesity indices, including WC, NC, WHR, LAP, VAI and CVAI, were divided into quartiles. We calculated the associations between the quartiles of abdominal obesity indices and the diabetic complications (Additional file 2: Tables S2–S4). Finally, we performed analyses on the BMI subgroups to investigate the associations of CVAI and NC with CVD and DKD among men and women (Additional file 3: Figure S2).

## Results

### General characteristics of the diabetic participants

Overall, 2144 men and 2514 women with diabetes were involved in the basal analyses. Among men, the average age was 67.4 ± 8.64 years, the prevalence of CVD was 35.5%, the prevalence of DKD was 24.9% and the prevalence of DR was 17.8%. In women, the average age was 66.9 ± 8.58 years, the prevalence of CVD was 38.3%, the prevalence of DKD was 25.4% and the prevalence of DR was 16.4%.

### Respective characteristics of men and women by diabetic complications

Tables 1 and 2 show the sociodemographic and general characteristics of the male and female participants in the study. The participants were divided into two groups with or without CVD, with or without DKD, and with or without DR, respectively. Compared with the men without CVD, BMI, WC, NC, HC, WHR and CVAI were significantly higher in men with CVD (all *P* < 0.05). However, no differences in LAP and VAI were found between the two groups (*P* > 0.05). BMI, WC, NC, HC, WHR, LAP, VAI and CVAI were all significantly higher in men with DKD than in men without DKD (all *P* < 0.05). No differences in BMI, WC, NC, HC, WHR, LAP, VAI and CVAI were found in men with or without DR (*P* > 0.05). Compared with the women without CVD, BMI, WC, NC, HC, WHR, LAP, VAI, CVAI were significantly higher in the women with CVD (all *P* < 0.05). BMI, WC, NC, HC, WHR, LAP, VAI, CVAI were significantly higher in women with DKD than in the women without DKD (all *P* < 0.05). Compared with the women without DR, BMI, WC and NC were significantly higher in female participants with DR (all *P* < 0.05).

**Table 1:** General characteristics of all male participants by diabetic complications

Characteristic	Diabetic complications								
	CVD−	CVD+	*P*	DKD−	DKD+	*P*	DR−	DR+	*P*
*N*	1373	755	–	1478	471	–	1224	265	–
Age, year	65.97 ± 8.65	70.11 ± 7.97	< 0.001	66.97 ± 8.39	68.89 ± 8.94	< 0.001	67.55 ± 8.68	67.06 ± 8.14	0.401
Duration of diabetes, year	8 (3, 15)	10 (5, 18)	< 0.001	8 (3, 15)	10 (6, 18)	< 0.001	9 (4, 15)	10 (5, 18)	0.001
Current smoking, %	29.7	39.9	< 0.001	34.3	39.9	0.030	33.2	35.4	0.523
Beyond high school education, %	60.5	58.1	0.234	61.4	57.5	0.141	59.3	63.5	0.212
BMI, kg/m^2^	24.82 ± 3.37	25.31 ± 3.09	0.001	24.77 ± 3.25	25.6 ± 3.31	< 0.001	24.99 ± 3.29	24.97 ± 3.2	0.903
WC, cm	91.54 ± 9.25	93.7 ± 8.85	< 0.001	91.56 ± 8.82	94.57 ± 9.71	< 0.001	92.39 ± 9.15	92.43 ± 9.42	0.947
NC, cm	39.65 ± 3.14	40.12 ± 3.07	0.001	39.58 ± 3.07	40.57 ± 3.18	< 0.001	39.81 ± 3.08	40.04 ± 3.01	0.261
HC, cm	99.31 ± 6.97	100.53 ± 6.57	< 0.001	99.44 ± 6.75	100.66 ± 7.16	0.001	99.75 ± 6.86	99.86 ± 7.01	0.817
WHR	0.92 ± 0.06	0.93 ± 0.06	< 0.001	0.92 ± 0.06	0.94 ± 0.06	< 0.001	0.93 ± 0.06	0.92 ± 0.06	0.830
LAP	40.0 (24.2, 63.5)	40.6 (27.4, 63.4)	0.280	38.1 (23.5, 59.3)	45.2 (31.5, 72.0)	< 0.001	40.7 (25.0, 63.4)	39.1 (23.8, 59.7)	0.246
VAI	1.84 (1.17, 2.95)	1.95 (1.23, 2.88)	0.470	1.78 (1.14, 2.79)	2.01 (1.38, 3.32)	< 0.001	1.88 (1.20, 2.93)	1.83 (1.18, 2.72)	0.287
CVAI	129.72 ± 40.74	141.57 ± 38.26	< 0.001	130.1 ± 39.21	145.14 ± 41.01	< 0.001	134.42 ± 40.1	133.8 ± 42.05	0.819
FPG, mmol/L	7.83 ± 2.38	7.82 ± 2.39	0.904	7.53 ± 2.09	8.69 ± 2.92	< 0.001	7.79 ± 2.32	8.25 ± 2.66	0.004
HbA1c, %	7.56 ± 1.41	7.63 ± 1.41	0.303	7.41 ± 1.27	8.14 ± 1.65	< 0.001	7.52 ± 1.4	7.89 ± 1.47	< 0.001
TC, mmol/L	5.01 ± 1.04	4.47 ± 1.14	< 0.001	4.82 ± 1.06	4.86 ± 1.25	0.483	4.81 ± 1.08	4.84 ± 1.05	0.763
TG, mmol/L	1.48 (1.05, 2.18)	1.42 (1.05, 2.01)	0.129	1.41 (1.02, 2.04)	1.57 (1.15, 2.24)	< 0.001	1.36 (1.03, 1.97)	1.46 (1.05, 2.13)	0.133
HDL, mmol/L	1.13 ± 0.26	1.08 ± 0.24	< 0.001	1.12 ± 0.26	1.09 ± 0.25	0.013	1.11 ± 0.26	1.1 ± 0.24	0.866
LDL, mmol/L	3.14 ± 0.74	2.75 ± 0.81	< 0.001	3 ± 0.76	3.01 ± 0.85	0.827	3.01 ± 0.78	3.02 ± 0.76	0.741
CCA plaque, %	63.6	77.4	< 0.001	67.4	71.7	0.096	68.2	71.9	0.271
Ln ACR, mg/g	2.71 ± 1.26	2.96 ± 1.37	< 0.001	2.2 ± 0.65	4.6 ± 1.12	< 0.001	2.71 ± 1.27	3.02 ± 1.42	0.001
eGFR, mL/min per 1.73 m^2^	92.65 ± 16.32	86.04 ± 17.72	< 0.001	92.16 ± 14.7	85.96 ± 21.24	< 0.001	89.94 ± 17.32	91.27 ± 16.73	0.254
Systolic blood pressure, mmHg	142.84 ± 19.52	146.21 ± 18.67	< 0.001	142.14 ± 18.88	151.19 ± 19.16	< 0.001	143.21 ± 19.08	147.27 ± 20.26	0.002
Diastolic blood pressure, mmHg	80.73 ± 11	79.11 ± 11.33	0.002	80.05 ± 10.87	81.06 ± 12.04	0.112	80.13 ± 11.24	80.3 ± 12.13	0.821

The data are summarized as the mean ± SD or median (interquartile range) for continuous variables or as a numerical proportion for categorical variables

*CVD* cardiovascular and cerebrovascular disease, *DKD* diabetic kidney disease, *DR* diabetic retinopathy, *BMI* body mass index, *FPG* fasting plasma glucose, *HbA1c* glycated hemoglobin, *HDL* high-density lipoprotein, *LDL* low-density lipoprotein, *TG* triglycerides, *TC* total cholesterol, *CCA* common carotid artery, *Ln ACR* log-transformed albumin to creatinine ratio, *eGFR* estimated glomerular filtration rate, *NC* neck circumference, *HC* hip circumference, *WC* waist circumference, *VAI* visceral adiposity index, *LAP* lipid accumulation product, *CVAI* Chinese visceral adiposity index, *WHR* waist-to-hip ratio

**Table 2:** General characteristics of all female participants by diabetic complications

Characteristic	Diabetic complications								
	CVD−	CVD+	*P*	DKD−	DKD+	*P*	DR−	DR+	*P*
*N*	1536	952	–	1453	422	–	1431	279	–
Age, year	65.15 ± 8.6	69.69 ± 7.79	< 0.001	66.71 ± 8.16	68.85 ± 8.97	< 0.001	66.72 ± 8.28	66.51 ± 7.69	0.693
Duration of diabetes, year	7 (3, 14)	10 (5, 18)	< 0.001	8 (3, 15)	10 (5, 18)	< 0.001	8 (3, 15)	10 (4.75, 19)	< 0.001
Current smoking, %	2.6	2.2	0.687	2.6	1.9	0.477	2.5	2.5	0.999
Beyond high school education, %	49.3	38.4	< 0.001	45.8	38.0	0.005	45.6	45.3	0.948
BMI, kg/m^2^	24.72 ± 3.85	25.27 ± 3.77	< 0.001	24.59 ± 3.71	26.16 ± 4	< 0.001	24.79 ± 3.82	25.45 ± 3.89	0.009
WC, cm	87.81 ± 9.97	89.68 ± 9.67	< 0.001	87.6 ± 9.64	92.27 ± 10.6	< 0.001	88.12 ± 9.98	89.62 ± 10.14	0.023
NC, cm	35.9 ± 2.94	36.35 ± 3.09	< 0.001	35.9 ± 2.97	36.87 ± 2.9	< 0.001	35.93 ± 2.94	36.41 ± 3.12	0.014
HC, cm	97.55 ± 8.85	98.51 ± 7.96	0.006	97.51 ± 8.18	99.92 ± 10.1	< 0.001	97.66 ± 8.83	98.66 ± 8.37	0.071
WHR	0.9 ± 0.07	0.91 ± 0.07	0.001	0.9 ± 0.07	0.93 ± 0.08	< 0.001	0.9 ± 0.07	0.91 ± 0.07	0.249
LAP	47.5 (30.0, 73.0)	51.7 (31.7, 77.9)	0.004	44.3 (28.4, 66.1)	60.1 (38.5, 95.3)	< 0.001	48.8 (30.7, 75.4)	48.1 (30.7, 74.1)	0.853
VAI	2.53 (1.64, 3.92)	2.64 (1.76, 4.04)	0.011	2.40 (1.58, 3.61)	3.01 (1.94, 4.79)	< 0.001	2.60 (1.69, 4.04)	2.36 (1.60, 3.67)	0.053
CVAI	122.58 ± 34.66	135.87 ± 31.67	< 0.001	123.45 ± 33.45	143.5 ± 33.87	< 0.001	126.43 ± 34.09	129.66 ± 34.48	0.149
FPG, mmol/L	7.67 ± 2.46	7.78 ± 2.62	0.297	7.54 ± 2.3	8.53 ± 3.12	< 0.001	7.65 ± 2.36	8.22 ± 2.93	< 0.001
HbA1c, %	7.37 ± 1.39	7.45 ± 1.3	0.121	7.3 ± 1.28	7.84 ± 1.57	< 0.001	7.31 ± 1.25	7.68 ± 1.56	< 0.001
TC, mmol/L	5.5 ± 1.16	5.13 ± 1.26	< 0.001	5.34 ± 1.18	5.44 ± 1.31	0.141	5.37 ± 1.21	5.49 ± 1.23	0.122
TG, mmol/L	1.62 (1.14, 2.23)	1.62 (1.20, 2.29)	0.203	1.52 (1.10, 2.12)	1.85 (1.26, 2.52)	< 0.001	1.63 (1.18, 2.31)	1.49 (1.13, 2.17)	0.084
HDL, mmol/L	1.31 ± 0.3	1.26 ± 0.29	< 0.001	1.32 ± 0.31	1.24 ± 0.28	< 0.001	3.29 ± 0.86	3.39 ± 0.9	0.078
LDL, mmol/L	3.39 ± 0.83	3.12 ± 0.91	< 0.001	3.27 ± 0.86	3.33 ± 0.92	0.286	1.29 ± 0.31	1.31 ± 0.29	0.382
CCA plaque, %	40.6	52.0	< 0.001	42.0	52.2	< 0.001	42.3	48.2	0.074
Ln ACR, mg/g	2.8 ± 1.12	2.94 ± 1.26	0.005	2.27 ± 0.64	4.47 ± 1.04	< 0.001	2.76 ± 1.14	3.04 ± 1.24	< 0.001
eGFR, mL/min per 1.73 m^2^	95.87 ± 15.19	88.13 ± 17.66	< 0.001	94.15 ± 14.54	89.47 ± 20.51	< 0.001	92.87 ± 16.33	94.23 ± 16.1	0.200
Systolic blood pressure, mmHg	144.73 ± 19.71	147.46 ± 20.19	0.001	143.34 ± 18.84	155.09 ± 20.86	< 0.001	144.98 ± 19.82	149.05 ± 19.97	0.002
Diastolic blood pressure, mmHg	78.35 ± 10.1	76.61 ± 10.68	< 0.001	76.76 ± 10.13	79.58 ± 11	< 0.001	77.35 ± 10.43	77.5 ± 9.27	0.819

The data are summarized as the mean ± SD or median (interquartile range) for continuous variables or as a numerical proportion for categorical variables

*CVD* cardiovascular and cerebrovascular disease, *DKD* diabetic kidney disease, *DR* diabetic retinopathy, *BMI* body mass index, *FPG* fasting plasma glucose, *HbA1c* glycated hemoglobin, *HDL* high-density lipoprotein, *LDL* low-density lipoprotein, *TG* triglycerides, *TC* total cholesterol, *CCA* common carotid artery, *Ln ACR* log-transformed albumin to creatinine ratio, *eGFR* estimated glomerular filtration rate, *NC* neck circumference, *HC* hip circumference, *WC* waist circumference, *VAI* visceral adiposity index, *LAP* lipid accumulation product, *CVAI* Chinese visceral adiposity index, *WHR* waist-to-hip ratio

### Associations between abdominal obesity indices and the prevalence of CVD

**Fig. 1:**
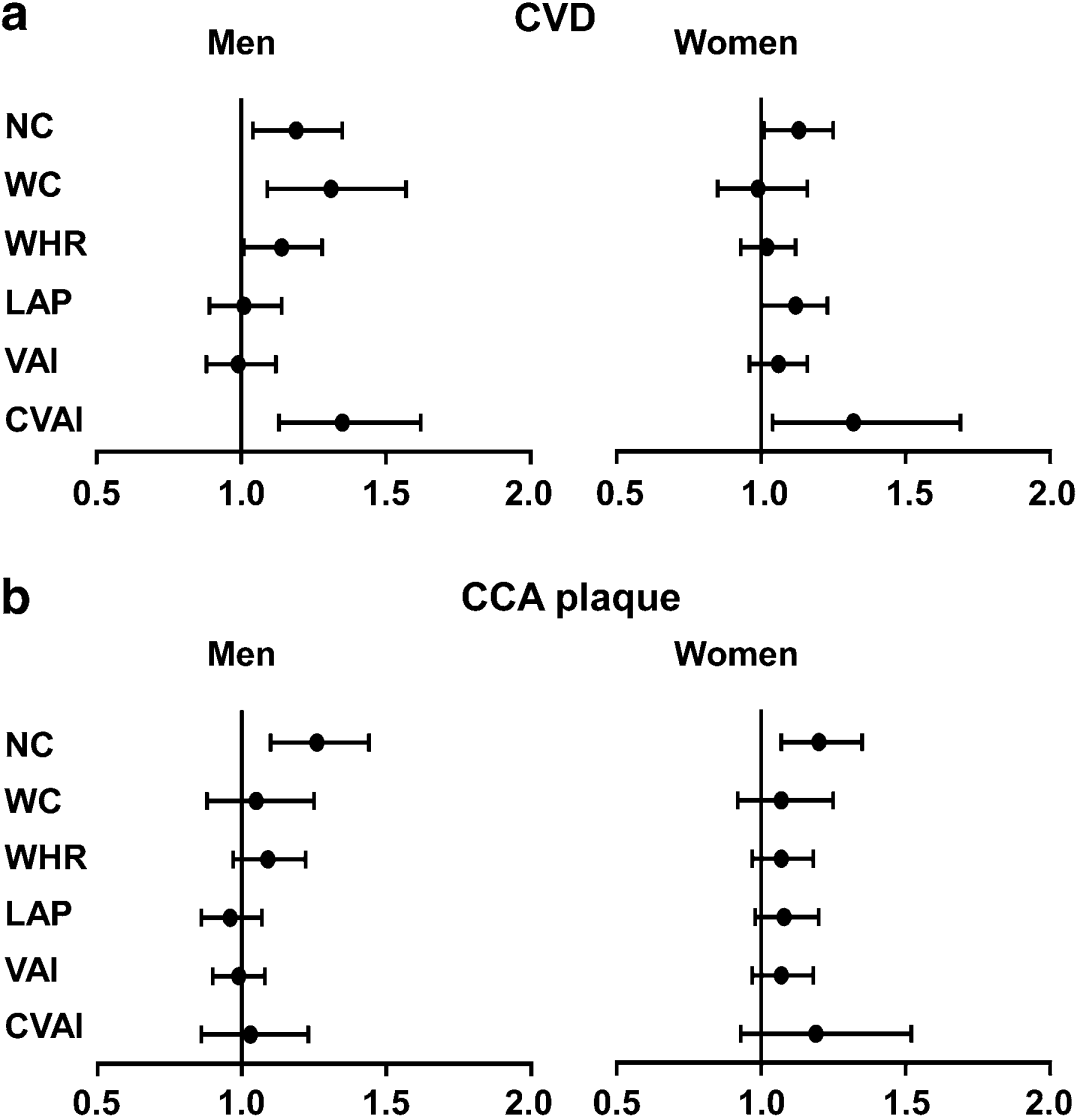
Associations between abdominal obesity indices and the prevalence of CVD. **a** Associations between abdominal obesity indices and the prevalence of CVD. **b** Associations between abdominal obesity indices and the prevalence of CCA plaque. Logistic regression analyses were used for the association of abdominal obesity indices with the prevalence of CVD and CCA plaque. The model was adjusted for age, education, duration of diabetes, current smoking, BMI, HbA1c, LDL and systolic blood pressure. *CVD* cardiovascular and cerebrovascular disease, *CCA* common carotid artery, *BMI* body mass index, *HbA1c* glycated hemoglobin, *NC* neck circumference, *VAI* visceral adiposity index, *LAP* the lipid accumulation product, *WC* waist circumference, *CVAI* Chinese visceral adiposity index, *WHR* waist-to-hip ratio, *LDL* low-density lipoprotein

Figure 1 indicated that increased NC and CVAI were significantly associated with greater prevalence of CVD both in men and women. Only NC, however, was significantly associated with a greater prevalence of CCA plaque.

In men, one SD increase in NC (OR 1.19; 95% CI 1.04, 1.35), WC (OR 1.31; 95% CI 1.09, 1.57), WHR (OR 1.14; 95% CI 1.01, 1.28) and CVAI (OR 1.35; 95% CI 1.13, 1.62) levels was significantly associated with a greater prevalence of CVD (*P* < 0.05). In women, a one SD increase in NC (OR 1.13; 95% CI 1.01, 1.97) and CVAI (OR 1.32; 95% CI 1.04, 1.69) levels was significantly associated with a greater prevalence of CVD (*P* < 0.05). Furthermore, a one SD increase in NC was significantly associated with a greater prevalence of CCA plaque both in men (OR 1.26; 95% CI 1.10, 1.44) and women (OR 1.20; 95% CI 1.07, 1.35). These associations were all fully adjusted for age, education, duration of diabetes, current smoking, BMI, HbA1c, systolic blood pressure and LDL.

### Associations between abdominal obesity indices and the prevalence of DKD

**Fig. 2:**
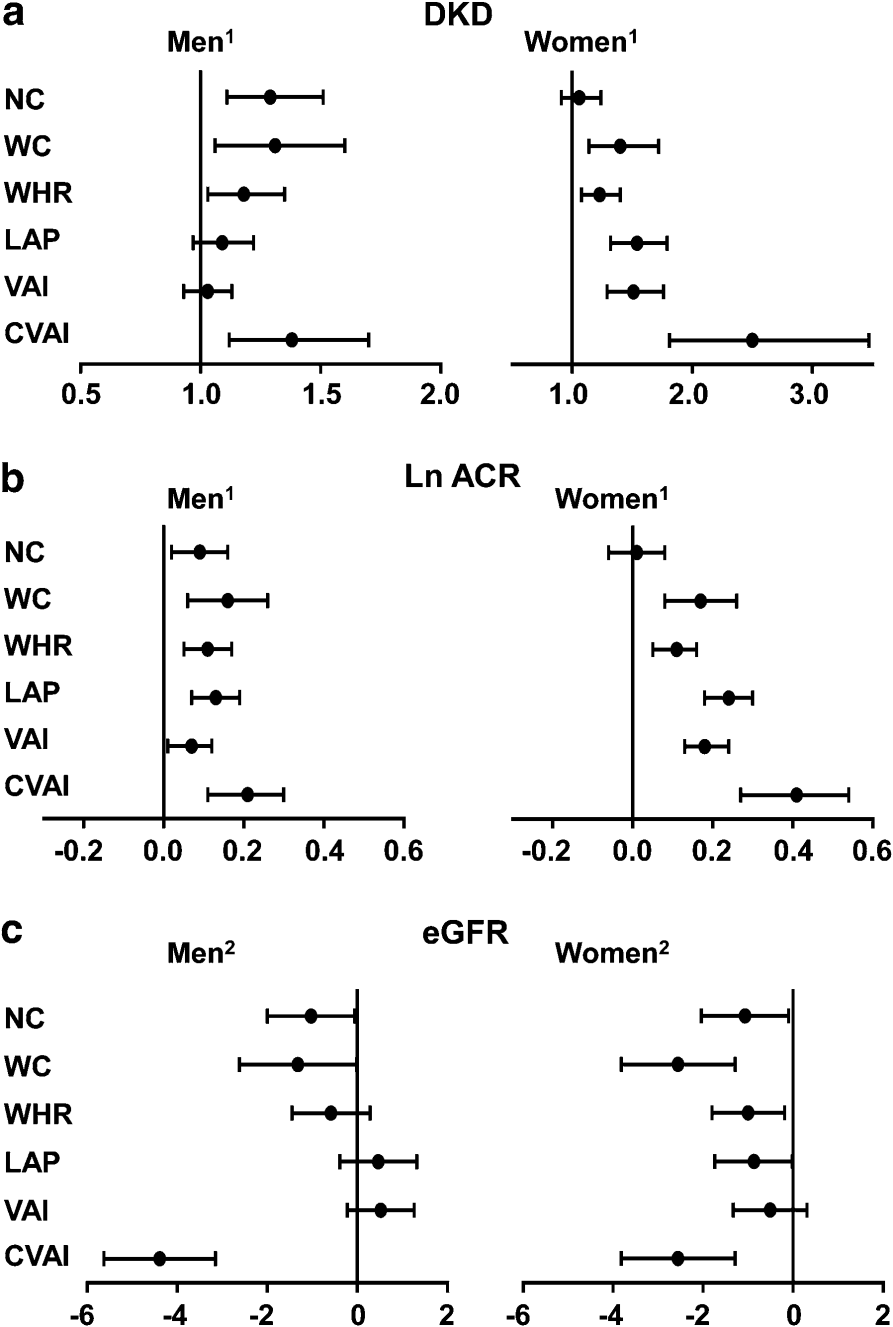
Associations between abdominal obesity indices and the prevalence of DKD. **a** Associations between abdominal obesity indices and the prevalence of DKD. **b** Associations between abdominal obesity indices and Ln ACR level. **c** Associations between abdominal obesity indices and eGFR level. Logistic regression analyses were used for the association of the abdominal obesity index with DKD. Linear regression analysis was used for the associations of the abdominal obesity index with Ln ACR and eGFR, respectively. ^1^The model was adjusted for age, education, duration of diabetes, current smoking, BMI, HbA1c, LDL and systolic blood pressure. ^2^The model was adjusted for education, duration of diabetes, current smoking, BMI, HbA1c, LDL and systolic blood pressure. *Ln ACR* log-transformed albumin to creatinine ratio; *eGFR* estimated glomerular filtration rate, *DKD* diabetic kidney disease, *BMI* body mass index, *HbA1c* glycated hemoglobin, *NC* neck circumference, *VAI* visceral adiposity index, *LAP* the lipid accumulation product, *WC* waist circumference, *CVAI* Chinese visceral adiposity index, *WHR* waist-to-hip ratio, *LDL* low-density lipoprotein

Figure 2 shows that increased WC, WHR and CVAI were significantly associated with a greater prevalence of DKD both in men and women. However, increased NC was significantly associated with a greater prevalence of DKD in men but not in women.

In men, a one SD increase in NC (OR 1.29; 95% CI 1.11, 1.51), WC (OR 1.31; 95% CI 1.06, 1.60), WHR (OR 1.18; 95% CI 1.03, 1.35) and CVAI (OR 1.38; 95% CI 1.12, 1.70) levels was significantly associated with a greater prevalence of DKD (*P* < 0.05). In women, a one SD increase in WC (OR 1.40; 95% CI 1.14, 1.72), WHR (OR 1.23; 95% CI 1.08, 1.40), LAP (OR 1.54; 95% CI 1.32, 1.79), VAI (OR 1.51; 95% CI 1.29, 1.76) and CVAI (OR 2.50; 95% CI 1.81, 3.47) levels was significantly associated with a greater prevalence of DKD (*P* < 0.05). Furthermore, a one SD increase in WC, WHR, LAP, VAI, CVAI levels were all significantly associated with higher ACR levels both in men and women (all *P* < 0.05). However, a one SD increase in NC was significantly associated with a higher ACR level in men (β 0.09, 95% CI 0.02, 0.16) (*P* < 0.05) rather than in women (β 0.05, 95% CI − 0.14, 0.24) (*P* > 0.05). These associations were all fully adjusted for age, education, duration of diabetes, current smoking, BMI, HbA1c, systolic blood pressure and LDL.

For the associations between the abdominal obesity indices and eGFR, Fig. 2c shows that a one SD increase in NC, WC, CVAI levels was significantly associated with decreased eGFR both in men and women (all *P* < 0.05). These associations were fully adjusted for education, duration of diabetes, current smoking, BMI, HbA1c, LDL and systolic blood pressure.

### Associations between abdominal obesity indices and the prevalence of DR

**Fig. 3:**
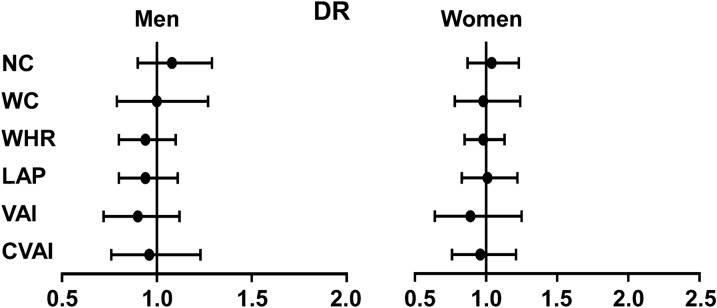
Associations between abdominal obesity indices and the prevalence of DR. Logistic regression analyses were used for the association between the abdominal obesity index and the prevalence of DR. The model was adjusted for age, sex, education, duration of diabetes, current smoking, BMI, HbA1c, LDL and systolic blood pressure. *DR* diabetic retinopathy, *BMI* body mass index, *HbA1c* glycated hemoglobin, *NC* neck circumference, *VAI* visceral adiposity index, *LAP* the lipid accumulation product, *WC* waist circumference, *CVAI* Chinese visceral adiposity index, *WHR* waist-to-hip ratio, *LDL* low-density lipoprotein

After adjusting for age, education, duration of diabetes, current smoking, BMI, HbA1c, LDL and systolic blood pressure, in both men and women, a one SD increase in NC, WC, WHR, LAP, VAI, and CVAI was not associated with the prevalence of DR (all *P* for trend > 0.05) (Fig. 3).

### Receiver-operating characteristics (ROC) curve analysis

**Fig. 4:**
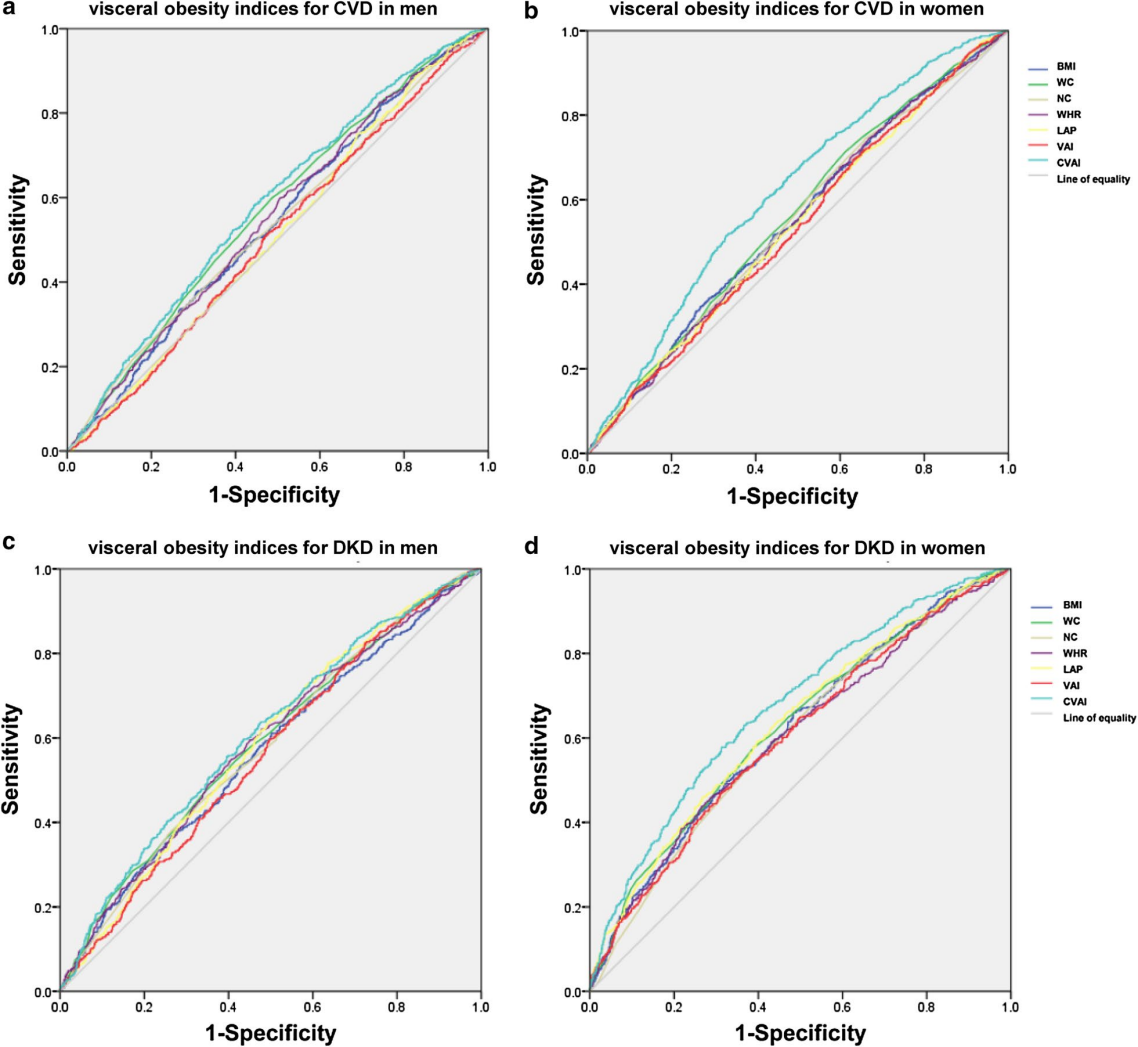
ROC curve of abdominal obesity indices for diagnosing CVD and DKD in men and women. **a** The ROC curve of abdominal obesity indices for diagnosing CVD in men. **b** The ROC curve of abdominal obesity indices for diagnosing CVD in women. **c** The ROC curve of abdominal obesity indices for diagnosing DKD in men. **d** The ROC curve of abdominal obesity indices for diagnosing DKD in women. *ROC* Receiver operating characteristic, *CVD* cardiovascular and cerebrovascular disease, *DKD* diabetic kidney disease, *NC* neck circumference, *VAI* visceral adiposity index, *LAP* lipid accumulation product, *WC* waist circumference, *CVAI* Chinese visceral adiposity index, *WHR* waist-to-hip ratio, *BMI* body mass index

Figure 4 shows the diagnostic ability of abdominal obesity indices including BMI, WC, NC, WHR, LAP, VAI and CVAI for CVD and DKD among men and women, respectively, analyzed by ROC curve. The differences between the area under the curve of CVAI and that of BMI, WC, NC, WHR, LAP and VAI for CVD and DKD among men and women were all significant (*P *< 0.01).

In men, area under ROC curve of BMI, WC, NC, WHR, LAP, VAI and CVAI for CVD was 0.543 (*P *< 0.01), 0.570 (*P *< 0.01), 0.541 (*P *< 0.01), 0.553 (*P *< 0.01), 0.514 (*P *= 0.273), 0.509 (*P *= 0.470) and 0.585 (*P *< 0.01), respectively (Fig. 1a). CVAI had the biggest area under the ROC curve compared to the other abdominal obesity indices, and the cutoff with the biggest Youden index of CVAI was 133.82 with a sensitivity of 58.3% and a specificity of 55.5%. The area under the ROC curve of BMI, WC, NC, WHR, LAP, VAI and CVAI for DKD was 0.568, 0.588, 0.580, 0.585, 0.585, 0.561 and 0.607, respectively (all *P *< 0.01) (Fig. 1c). CVAI had the biggest area under the ROC curve compared to the other abdominal obesity indices, and the cutoff with the biggest Youden index of CVAI was 131.67 with a sensitivity of 63.6% and a specificity of 52.5%.

In women, the area under ROC curve of BMI, WC, NC, WHR, LAP, VAI and CVAI for CVD was 0.549, 0.558, 0.546, 0.542, 0.536, 0.531 and 0.617, respectively (all *P *< 0.01) (Fig. 1b). CVAI had the biggest area under the ROC curve compared to other abdominal obesity indices, and the cutoff with the biggest Youden index of CVAI was 135.09 with a sensitivity of 51.5% and a specificity of 67.1%. The area under the ROC curve of BMI, WC, NC, WHR, LAP, VAI and CVAI for CVD was 0.617, 0.627, 0.604, 0.604, 0.633, 0.603 and 0.675, respectively (all *P *< 0.01) (Fig. 1d). CVAI had the biggest area under the ROC curve compared to the other abdominal obesity indices, and the cutoff with the biggest Youden index of CVAI was 137.11 with a sensitivity of 57.8% and a specificity of 68.7%.

### Sensitivity analyses

Additional file 2: Table S1 showed that in men, increased BMI, NC, WC, WHR, LAP, VAI and CVAI level was associated with a higher prevalence of CVD and DKD (all *P* for trend < 0.05). Higher CVAI by a one SD was significantly associated with a 29% and 42% (higher than the other abdominal obesity indices) increased prevalence of CVD and DKD events, respectively. In women, increased BMI, NC, WC, LAP, VAI and CVAI level was associated with a higher prevalence of CVD and DKD (all *P* for trend < 0.05). Higher CVAI by a one SD was significantly associated with a 27% and 75% (higher than the other abdominal obesity indices) increased prevalence of CVD and DKD events, respectively. However, no significant associations of BMI, NC, WC, WHR, LAP, VAI, and CVAI with the prevalence of DR among men or women were found.

The association between the quartiles of NC and the prevalence of CVD remained significant in men, rather than in women, although the associations between the quartiles of NC and the prevalence of CCA plaque remained significant in both men and women (Additional file 2: Table S2). The associations between the quartiles of CVAI and the prevalence of CVD remained significant in both men and women (Additional file 2: Table S2). Increased NC was still significantly associated with a greater prevalence of DKD in men, rather than in women, although increased CVAI was significantly associated with a greater prevalence of DKD in both men and women (Additional file 2: Table S3). The quartiles of NC, WC, WHR, LAP, VAI, and CVAI were not associated with the prevalence of DR in either men or women (Additional file 2: Table S4).

In subgroups of BMI analyses, larger CVAI and NC were associated with a higher prevalence of CVD and DKD in participants without obesity (BMI < 28 kg/m^2^); however, no significant associations of CVAI and NC with the prevalence of CVD and DKD were found in participants with obesity (BMI > 28 kg/m^2^) (Additional file 3: Figure S2).

## Discussion

The influence of visceral adiposity on the development of diabetes has been reported previously.

Higher visceral fat area at baseline has been considered an independent risk factor for developing T2DM, and the optimal visceral fat area cutoff values are markedly different between men and women [32]. Visceral fat mass is positively related to plasma sphingosine-1-phosphate, fibroblast growth factor 23, and neutrophil gelatinase-associated lipocalin levels [33, 34], while serum osteocalcin and serum meteorin-like levels inversely correlate with visceral fat mass [34, 35]. Liraglutide decreases visceral adipose tissue volume and has been associated with improved glycemic control in South Asians [36]. However, studies focused on the associations of abdominal obesity indices with diabetic complications, especially the NC and CVAI, are limited.

In the present study, we found that CVAI had the strongest associations with the prevalence of CVD and DKD among these abdominal obesity indices; NC had a unique association with the prevalence of CCA plaque. All the associations were independent of BMI. However, all the abdominal obesity indices were not associated with the prevalence of DR, regardless of adjustment for BMI or not. To the best of our knowledge, this is the first study to evaluate the relationships between NC, WC, WHR, LAP, VAI, CVAI and the prevalence of CVD, DKD and DR simultaneously. The different associations between the abdominal obesity indices and the prevalence of CVD, DKD and DR may partly account for the different incidence rates of diabetic complications. In addition, calculating CVAI in diabetic adults and improving the abnormal distribution of adipose tissue in a timely fashion might be helpful for both prevention and intervention of CVD and DKD.

### Consistent associations of CVAI with CVD and DKD both in men and women

CVAI is a novel visceral adiposity index developed in Chinese adults that is associated with visceral fat area and insulin resistance [12, 37]. In our study, only CVAI was associated with CVD and DKD independently of BMI both in men and women, while NC, WC and WHR were associated with CVD independently of BMI in men but not in women. In women, NC, WC, WHR and LAP were significantly associated with CVD without adjusting for BMI (Additional file 2: Table S1). One reason for this finding may be that fat distribution differs between men and women; apple-shaped obesity is more common in men and pear-shaped obesity in women [1]. The other reason may be that NC, WC, WHR and LAP only reflect fat mass in the abdominal region without distinguishing between visceral and subcutaneous fat mass, however CVAI reflects visceral fat mass [37]. The gender differences in the associations of NC, WC, WHR, LAP with CVD and the gender consistency in the associations of CVAI with CVD suggested that CVAI may be more suitable and convenient for the prevention and control of CVD than other abdominal obesity indices in adults with diabetes.

### Stronger associations of CVAI with CVD and DKD than BMI

Previous studies reported that CVAI was superior to BMI, WC or VAI for the diagnosis of diabetes and prediabetes [12, 13, 37], which is similar to the results of our study. In our study, the area under the ROC curve of CVAI for the diagnosis of CVD and DKD was largest, and the OR per 1 SD increase in CVAI with CVD and DKD was highest among NC, WC, WHR, LAP, VAI, and CVAI both in men and women, which suggested that CVAI had the strongest association with CVD and DKD among the abdominal obesity indices, independent of BMI. Compared with BMI, the area under the ROC curve of CVAI for the diagnosis of CVD and DKD was larger, and the OR per 1 SD increase in CVAI with CVD and DKD was higher when adjusting for the same confounders (Additional file 2: Table S1), which supported that abdominal obesity is more closely associated with CVD and DKD compared with generalized obesity [9, 17, 38]. Furthermore, in the subgroup analyses, we found that the positive association of CVAI with CVD and DKD among men and women without obesity remained significant, indicating that combining BMI with CVAI for the prevention and treatment of diabetes may be a beneficial approach.

### The unique association between NC and the prevalence of CCA plaque

NC has been considered a marker of upper body subcutaneous fat deposits and a simple and valuable screening tool for identifying individuals with obesity [11, 14]. Studies have reported that NC is independently associated with hyperuricemia [39], non-alcoholic fatty liver disease [40], metabolic syndrome [14, 41] and obstructive sleep apnea [42]. Furthermore, cross-sectional and prospective cohort studies suggested that large NC values may be associated with cardiovascular risk factors, even all-cause mortality, in both men and women [43–45], which was similar to the results found in our study. Interestingly, we further found that a larger NC was not only independently associated with a higher prevalence of CVD but also CCA plaque, which has been considered a strong predictor of cardiovascular outcomes [46]. However, not all of the other abdominal obesity indices, including WC, WHR, LAP, VAI and CVAI were significantly associated with the prevalence of CCA plaque. These findings may indicate that the harmful effects of large NC on the kidneys may start in the early stages in patients with diabetes. Lipolytic activity may be one of the mechanisms underlying NC with CVD. It has been demonstrated that free fatty acids released from upper body subcutaneous fat, which result in oxidative stress and vascular injury [47], were more harmful than free fatty acids released from lower body subcutaneous fat [48]. However, much of the pathogenesis of NC and CVD remains unknown.

This study has several strengths. First, it is the first study to detect the associations of obesity phenotype indices with CVD, DKD and DR concurrently. Second, the participants were enrolled from a community with a relatively large sample size, and thus the results may be more reflective of the general population of diabetic individuals. Third, anthropometric measurements and questionnaires were administered by the same trained research group, ensuring the quality of the data. However, there are also some limitations in our study. First, being a cross-sectional study, causal inference between obesity phenotype indices and diabetic complications cannot be established. Second, the ethnic group investigated was only Han Chinese, thus generalizing the results to other ethnic groups should be done cautiously. Third, we did not test for oxidative stress markers in the initial design. Further testing the oxidative stress markers such as malondialdehyde and advanced oxidation protein product levels in the plasma samples of visceral obesity patients should be considered in the future.

## Conclusions

The present study demonstrates that CVAI had the strongest association with the prevalence of CVD and DKD among the abdominal obesity indices, and NC had a unique association with the prevalence of CCA plaque. CVAI might be a useful and powerful tool for the prevention and treatment of CVD and DKD, and NC may be a convenient and valuable anthropometric measurement for early prevention of CVD. Further prospective studies are necessary to examine our findings in external populations.

## Supplementary information

**Additional file 1: Figure S1.** Flowchart of sampling frame and participants.

**Additional file 2: Table S1.** Associations between adiposity phenotype indices and the prevalence of diabetic complications without adjusting for BMI. **Table S2.** Associations between the quartiles of the abdominal obesity indices and the prevalence of CVD. **Table S3.** Associations between the quartiles of the abdominal obesity indices and the prevalence of DKD. **Table S4.** Associations between the quartiles of the abdominal obesity indices and the prevalence of DR.

**Additional file 3: Figure S2.** The associations of CVAI with CVD and DKD in different subgroups of BMI. Analyses were stratified by BMI. The black dots represent ORs, and the horizontal lines represent 95% confidence intervals. CVD, cardiovascular and cerebrovascular disease; CVAI, Chinese visceral adiposity index; BMI, body mass index; OR, odds ratio.

## Data Availability

The data supporting the findings of this study are available upon reasonable request from the corresponding authors.

## Abbreviations

T2DM: Type 2 diabetes mellitus
CVD: Cardiovascular and cerebrovascular disease
DKD: Diabetic kidney disease
DR: Diabetic retinopathy
BMI: Body mass index
FPG: Fasting plasma glucose
HbA1c: Glycated hemoglobin
HDL: High-density lipoprotein
LDL: Low-density lipoprotein
TG: Triglycerides
TC: Total cholesterol
CCA: Common carotid artery
Ln ACR: Log-transformed albumin to creatinine ratio
eGFR: Estimated glomerular filtration rate
NC: Neck circumference
HC: Hip circumference
WC: Waist circumference
VAI: Visceral adiposity index
LAP: Lipid accumulation product
CVAI: Chinese visceral adiposity index
WHR: Waist-to-hip ratio
OR: Odds ratio
CIs: Confidence intervals
SD: Standard deviation
