# Supplementary material for: Associations between abdominal obesity indices and diabetic complications: Chinese visceral adiposity index and neck circumference
Source: Cardiovasc Diabetol. 2020 Jul 31;19:118. doi: 10.1186/s12933-020-01095-4 (PMC7395356; doi:10.1186/s12933-020-01095-4)
Supplement: Supplementary file 2 — Additional file 2: Table S1. Associations between adiposity phenotype indices and the prevalence of diabetic complications without adjusting for BMI. Table S2. Associations between the quartiles of the abdominal obesity indices and the prevalence of CVD. Table S3. Associations between the quartiles of the abdominal obesity indices and the prevalence of DKD. Table S4. Associations between the quartiles of the abdominal obesity indices and the prevalence of DR. [file 12933_2020_1095_MOESM2_ESM.docx]

Table S1 Associations between adiposity phenotypes indices and the prevalence of diabetic complications without adjusting for BMI

|  | | Men | | | Women | | |
| --- | --- | --- | --- | --- | --- | --- | --- |
|  |  | CVD | DKD | DR | CVD | DKD | DR |
| NC | Quartile 1 | Ref. | Ref. | Ref. | Ref. | Ref. | Ref. |
|  | Quartile 2 | 1.08 (0.83, 1.40) | 1.37 (1.00, 1.89) | 1.25 (0.87, 1.80) | 1.62 (1.27, 2.05) | 1.54 (1.10, 2.16) | 1.04 (0.73, 1.50) |
|  | Quartile 3 | 1.25 (0.94, 1.66) | 1.92 (1.36, 2.70) | 1.11 (0.74, 1.68) | 1.41 (1.08, 1.84) | 1.72 (1.20, 2.47) | 1.05 (0.71, 1.56) |
|  | Quartile 4 | 1.72 (1.28, 2.31) | 2.10 (1.49, 2.96) | 1.12 (0.74, 1.70) | 1.71 (1.31, 2.23) | 2.11 (1.48, 3.02) | 1.41 (0.96, 2.07) |
|  | *P* for trend | <0.001 | <0.001 | 0.667 | <0.001 | <0.001 | 0.112 |
|  | per SD increase | 1.25 (1.12, 1.38) | 1.40 (1.24, 1.58) | 1.01 (0.87, 1.16) | 1.19 (1.09, 1.31) | 1.30 (1.15, 1.47) | 1.11 (0.97, 1.27) |
| WC | Quartile 1 | Ref. | Ref. | Ref. | Ref. | Ref. | Ref. |
|  | Quartile 2 | 1.20 (0.90, 1.61) | 1.01 (0.71, 1.44) | 1.14 (0.78, 1.68) | 1.29 (1.00, 1.67) | 1.11 (0.76, 1.61) | 0.96 (0.65, 1.43) |
|  | Quartile 3 | 1.66 (1.25, 2.22) | 1.51 (1.07, 2.13) | 0.72 (0.47, 1.09) | 1.31 (1.01, 1.69) | 1.52 (1.07, 2.17) | 1.08 (0.73, 1.61) |
|  | Quartile 4 | 1.73 (1.29, 2.32) | 2.07 (1.47, 2.90) | 0.92 (0.61, 1.37) | 1.48 (1.14, 1.91) | 2.48 (1.76, 3.51) | 1.35 (0.91, 2.01) |
|  | *P* for trend | <0.001 | <0.001 | 0.265 | 0.005 | <0.001 | 0.101 |
|  | per SD increase | 1.27 (1.15, 1.42) | 1.38 (1.22, 1.57) | 0.95 (0.83, 1.10) | 1.15 (1.05, 1.27) | 1.53 (1.34, 1.73) | 1.11 (0.97, 1.28) |
| WHR | Quartile 1 | Ref. | Ref. | Ref. | Ref. | Ref. | Ref. |
|  | Quartile 2 | 1.32 (0.98, 1.76) | 1.10 (0.76, 1.58) | 0.90 (0.61, 1.34) | 1.16 (0.90, 1.51) | 0.91 (0.62, 1.32) | 0.85 (0.58, 1.25) |
|  | Quartile 3 | 1.51 (1.13, 2.02) | 1.80 (1.27, 2.55) | 0.75 (0.50, 1.13) | 1.20 (0.93, 1.57) | 1.42 (0.99, 2.05) | 0.91 (0.62, 1.35) |
|  | Quartile 4 | 1.77 (1.32, 2.37) | 1.74 (1.23, 2.46) | 0.83 (0.56, 1.24) | 1.17 (0.90, 1.53) | 1.92 (1.35, 2.73) | 0.83 (0.56, 1.23) |
|  | *P* for trend | <0.001 | <0.001 | 0.256 | 0.234 | <0.001 | 0.443 |
|  | per SD increase | 1.20 (1.08, 1.34) | 1.29 (1.14, 1.46) | 0.92 (0.80, 1.07) | 1.07 (0.98, 1.18) | 1.35 (1.19, 1.52) | 1.02 (0.89, 1.17) |
| LAP | Quartile 1 | Ref. | Ref. | Ref. | Ref. | Ref. | Ref. |
|  | Quartile 2 | 1.56 (1.17, 2.08) | 1.41 (0.98, 2.04) | 0.90 (0.61, 1.33) | 1.27 (0.98, 1.65) | 1.32 (0.89, 1.95) | 1.00 (0.68, 1.47) |
|  | Quartile 3 | 1.63 (1.22, 2.18) | 1.84 (1.29, 2.63) | 0.85 (0.57, 1.26) | 1.40 (1.07, 1.82) | 1.66 (1.14, 2.42) | 0.91 (0.61, 1.34) |
|  | Quartile 4 | 1.85 (1.37, 2.51) | 2.45 (1.71, 3.51) | 0.72 (0.47, 1.09) | 1.53 (1.17, 1.99) | 3.25 (2.24, 4.70) | 0.78 (0.52, 1.17) |
|  | *P* for trend | <0.001 | <0.001 | 0.118 | 0.002 | <0.001 | 0.200 |
|  | per SD increase | 1.08 (0.97, 1.20) | 1.18 (1.05, 1.32) | 0.93 (0.79, 1.08) | 1.17 (1.06, 1.29) | 1.70 (1.47, 1.97) | 0.87 (0.74, 1.02) |
| VAI | Quartile 1 | Ref. | Ref. | Ref. | Ref. | Ref. | Ref. |
|  | Quartile 2 | 1.18 (0.88, 1.57) | 1.87 (1.31, 2.67) | 1.03 (0.70, 1.53) | 1.56 (1.20, 2.03) | 1.30 (0.90, 1.90) | 0.93 (0.62, 1.40) |
|  | Quartile 3 | 1.73 (1.30, 2.31) | 1.66 (1.15, 2.38) | 1.06 (0.72, 1.58) | 1.56 (1.20, 2.04) | 1.60 (1.11, 2.30) | 1.27 (0.86, 1.88) |
|  | Quartile 4 | 1.55 (1.15, 2.09) | 2.41 (1.69, 3.44) | 0.72 (0.47, 1.09) | 1.54 (1.18, 2.02) | 2.23 (1.56, 3.20) | 1.29 (0.87, 1.90) |
|  | *P* for trend | <0.001 | <0.001 | 0.168 | 0.003 | <0.001 | 0.086 |
|  | per SD increase | 1.01 (0.91, 1.13) | 1.05 (0.95, 1.16) | 0.88 (0.71, 1.10) | 1.07 (0.97, 1.18) | 1.56 (1.33, 1.82) | 1.11 (0.97, 1.28) |
| CVAI | Quartile 1 | Ref. | Ref. | Ref. | Ref. | Ref. | Ref. |
|  | Quartile 2 | 1.27 (0.94, 1.71) | 1.43 (0.99, 2.07) | 1.04 (0.71, 1.54) | 1.36 (1.03, 1.78) | 1.20 (0.79, 1.80) | 0.87 (0.58, 1.32) |
|  | Quartile 3 | 1.81 (1.35, 2.42) | 1.79 (1.25, 2.57) | 0.65 (0.43, 0.99) | 1.60 (1.21, 2.11) | 1.76 (1.19, 2.61) | 1.19 (0.81, 1.77) |
|  | Quartile 4 | 1.84 (1.37, 2.47) | 2.66 (1.87, 3.78) | 0.91 (0.61, 1.36) | 1.88 (1.41, 2.51) | 3.23 (2.17, 4.80) | 1.32 (0.89, 1.94) |
|  | *P* for trend | <0.001 | <0.001 | 0.258 | <0.001 | <0.001 | 0.065 |
|  | per SD increase | 1.29 (1.16, 1.44) | 1.42 (1.25, 1.61) | 0.94 (0.81, 1.08) | 1.27 (1.14, 1.41) | 1.75 (1.51, 2.02) | 1.10 (0.96, 1.26) |
| BMI | Quartile 1 | Ref. | Ref. | Ref. | Ref. | Ref. | Ref. |
|  | Quartile 2 | 1.40 (1.04, 1.87) | 1.01 (0.72, 1.42) | 1.05 (0.70, 1.56) | 1.49 (1.15, 1.94) | 1.53 (1.06, 2.21) | 1.01 (0.68, 1.52) |
|  | Quartile 3 | 1.42 (1.06, 1.91) | 1.26 (0.90, 1.77) | 0.93 (0.62, 1.39) | 1.31 (1.00, 1.70) | 1.67 (1.17, 2.39) | 1.20 (0.81, 1.78) |
|  | Quartile 4 | 1.60 (1.19, 2.15) | 2.05 (1.47, 2.85) | 0.89 (0.59, 1.34) | 1.85 (1.43, 2.41) | 2.75 (1.94, 3.91) | 1.40 (0.95, 2.07) |
|  | *P* for trend | 0.003 | <0.001 | 0.457 | <0.001 | <0.001 | 0.056 |
|  | per SD increase | 1.21 (1.09, 1.34) | 1.33 (1.18, 1.50) | 0.94 (0.81, 1.09) | 1.21 (1.10, 1.33) | 1.51 (1.34, 1.71) | 1.15 (0.99, 1.32) |

2488 women were included to analyze the association between adiposity phenotypes index and CVD; 1875 women were included to analyze the association between adiposity phenotypes index and DKD; 1710 women were included to analyze the association between adiposity phenotypes index and DR. 2128 men were included to analyze the association between adiposity phenotypes index and CVD; 1949 men were included to analyze the association between adiposity phenotypes index and DKD; 1489 men were included to analyze the association between adiposity phenotypes index and DR.

Data are expressed as regression coefficients or odds ratios (95% CI). Logistic regression analyses were used for the association between adiposity phenotypes index and the prevalence of CVD, DKD and DR, respectively.

The model was adjusted for age, education, duration of diabetes history, current smoking, HbA1c, LDL and systolic blood pressure.

CVD, cardiovascular and cerebrovascular disease; DKD, diabetic kidney disease; DR, diabetic retinopathy; HbA1c, glycated hemoglobin; NC, neck circumference; VAI, visceral adiposity index; LAP, the lipid accumulation product; WC, waist circumference; CVAI, Chinese visceral adiposity index; WHR, waist-to-hip ratio; LDL, low density lipoprotein-cholesterol; BMI, body mass index.

Table S2 Associations between the quartiles of the abdominal obesity indices and the prevalence of CVD

|  |  | Men | | Women | |
| --- | --- | --- | --- | --- | --- |
|  |  | CVD | CCA plaque | CVD | CCA plaque |
| NC | Quartile 1 | Ref. | Ref. | Ref. | Ref. |
|  | Quartile 2 | 1.01 (0.77, 1.32) | 1.06 (0.81, 1.40) | 1.52 (1.18, 1.95) | 1.24 (0.97, 1.59) |
|  | Quartile 3 | 1.12 (0.82, 1.53) | 1.20 (0.87, 1.66) | 1.27 (0.95, 1.69) | 1.59 (1.19, 2.12) |
|  | Quartile 4 | 1.44 (1.01, 2.06) | 1.53 (1.05, 2.23) | 1.42 (1.02, 1.97) | 1.43 (1.03, 1.99) |
|  | *P* for trend | 0.049 | 0.029 | 0.082 | 0.008 |
| WC | Quartile 1 | Ref. | Ref. | Ref. | Ref. |
|  | Quartile 2 | 1.15 (0.85, 1.57) | 1.14 (0.84, 1.55) | 1.17 (0.89, 1.53) | 1.36 (1.04, 1.77) |
|  | Quartile 3 | 1.55 (1.10, 2.19) | 1.20 (0.85, 1.70) | 1.08 (0.80, 1.45) | 1.26 (0.93, 1.70) |
|  | Quartile 4 | 1.52 (1.01, 2.34) | 1.20 (0.77, 1.86) | 1.02 (0.70, 1.49) | 1.06 (0.72, 1.55) |
|  | *P* for trend | 0.022 | 0.382 | 0.983 | 0.665 |
| WHR | Quartile 1 | Ref. | Ref. | Ref. | Ref. |
|  | Quartile 2 | 1.24 (0.92, 1.67) | 1.27 (0.95, 1.71) | 1.09 (0.83, 1.41) | 0.97 (0.75, 1.27) |
|  | Quartile 3 | 1.38 (1.01, 1.87) | 1.28 (0.94, 1.74) | 1.08 (0.82, 1.41) | 1.00 (0.76, 1.31) |
|  | Quartile 4 | 1.54 (1.12, 2.13) | 1.21 (0.87, 1.67) | 1.01 (0.76, 1.33) | 1.10 (0.83, 1.45) |
|  | *P* for trend | 0.007 | 0.295 | 0.994 | 0.487 |
| LAP | Quartile 1 | Ref. | Ref. | Ref. | Ref. |
|  | Quartile 2 | 1.45 (1.07, 1.96) | 1.15 (0.84, 1.56) | 1.18 (0.90, 1.55) | 1.15 (0.88, 1.51) |
|  | Quartile 3 | 1.47 (1.07, 2.02) | 1.02 (0.74, 1.41) | 1.23 (0.92, 1.64) | 1.05 (0.79, 1.40) |
|  | Quartile 4 | 1.62 (1.14, 2.30) | 0.98 (0.69, 1.39) | 1.28 (0.94, 1.74) | 1.02 (0.75, 1.39) |
|  | *P* for trend | 0.014 | 0.688 | 0.129 | 0.929 |
| VAI | Quartile 1 | Ref. | Ref. | Ref. | Ref. |
|  | Quartile 2 | 1.10 (0.82, 1.47) | 1.10 (0.82, 1.48) | 1.49 (1.14, 1.94) | 1.01 (0.78, 1.32) |
|  | Quartile 3 | 1.59 (1.18, 2.13) | 1.11 (0.82, 1.50) | 1.44 (1.10, 1.88) | 1.39 (1.06, 1.82) |
|  | Quartile 4 | 1.40 (1.03, 1.90) | 0.98 (0.72, 1.33) | 1.43 (1.01, 1.88) | 0.99 (0.75, 1.30) |
|  | *P* for trend | 0.006 | 0.881 | 0.023 | 0.536 |
| CVAI | Quartile 1 | Ref. | Ref. | Ref. | Ref. |
|  | Quartile 2 | 1.24 (0.91, 1.71) | 1.04 (0.77, 1.42) | 1.34 (0.99, 1.81) | 1.08 (0.81, 1.46) |
|  | Quartile 3 | 1.75 (1.24, 2.47) | 1.03 (0.73, 1.46) | 1.54 (1.09, 2.20) | 1.13 (0.79, 1.60) |
|  | Quartile 4 | 1.74 (1.13, 2.68) | 1.02 (0.66, 1.57) | 1.73 (1.07, 2.81) | 1.01 (0.62, 1.64) |
|  | *P* for trend | 0.003 | 0.955 | 0.022 | 0.843 |

Data are expressed as regression coefficients (95%CI). Logistic regression analyses were used for the association of abdominal obesity indices with the prevalence of CVD and CCA plaque.

The model was adjusted for age, education, duration of diabetes history, current smoking, BMI, HbA1c, LDL and systolic blood pressure.

CVD, cardiovascular and cerebrovascular disease; CCA, common carotid artery; BMI, body mass index; HbA1c, glycated hemoglobin; NC, neck circumference; VAI, visceral adiposity index; LAP, the lipid accumulation product; WC, waist circumference; CVAI, Chinese visceral adiposity index; WHR, waist-to-hip ratio; LDL, low-density lipoprotein.

Table S3 Associations between the quartiles of the abdominal obesity indices and the prevalence of DKD

|  |  | Men | | | Women | | |
| --- | --- | --- | --- | --- | --- | --- | --- |
|  |  | DKD^1^ | Ln ACR^1^ | eGFR^2^ | DKD^1^ | Ln ACR^1^ | eGFR^2^ |
| NC | Q1 | Ref. | Ref. | Ref. | Ref. | Ref. | Ref. |
|  | Q2 | 1.24 (0.89, 1.73) | 0.01 (-0.15, 0.15) | -1.09 (-3.12, 0.93) | 1.30 (0.92, 1.84) | 0.03 (-0.12, 0.17) | 0.27 (-1.75, 2.30) |
|  | Q3 | 1.62 (1.12, 2.35) | 0.06 (-0.12, 0.23) | -1.67 (-3.05, 1.71) | 1.23 (0.83, 1.82) | -0.06 (-0.23, 0.11) | -2.01 (-4.36, 0.35) |
|  | Q4 | 1.58 (1.04, 2.41) | 0.17 (0.03, 0.37) | -1.87 (-4.59, -0.85) | 1.23 (0.79, 1.90) | 0.05 (-0.14, 0.24) | -2.62 (-5.33, -0.10) |
|  | *P* for trend | 0.014 | 0.011 | 0.046 | 0.441 | 0.924 | 0.024 |
| WC | Quartile 1 | Ref. | Ref. | Ref. | Ref. | Ref. | Ref. |
|  | Quartile 2 | 0.92 (0.63, 1.33) | -0.14 (-0.30, 0.03) | -1.68 (-3.93, 0.65) | 0.99 (0.67, 1.45) | -0.01 (-0.17, 0.15) | -0.882 (-3.08, 1.31) |
|  | Quartile 3 | 1.27 (0.85, 1.91) | 0.06 (-0.13, 0.25) | -3.76 (-6.30, -1.21) | 1.19 (0.80, 1.77) | -0.01 (-0.18, 0.16) | -2.49 (-4.89, -0.09) |
|  | Quartile 4 | 1.52 (1.02, 2.51) | 0.14 (0.01, 0.38) | -2.90 (-6.13, -0.32) | 1.59 (1.00, 2.59) | 0.27 (0.06, 0.49) | -4.30 (-7.34, -1.25) |
|  | *P* for trend | 0.042 | 0.045 | 0.028 | 0.049 | 0.048 | 0.004 |
| WHR | Quartile 1 | Ref. | Ref. | Ref. | Ref. | Ref. | Ref. |
|  | Quartile 2 | 0.97 (0.67, 1.41) | 0.06 (-0.10, 0.23) | -1.10 (-3.30, 1.10) | 0.80 (0.55, 1.18) | -0.09 (-0.25, 0.06) | 0.01 (-2.16, 2.16) |
|  | Quartile 3 | 1.50 (1.05, 2.16) | 0.19 (0.02, 0.36) | -1.08 (-3.35, 1.19) | 1.19 (0.82, 1.73) | 0.06 (-0.10, 0.22) | 0.28 (-1.95, 2.51) |
|  | Quartile 4 | 1.42 (1.01, 1.94) | 0.20 (0.02, 0.38) | -2.34 (-4.75, -0.06) | 1.44 (1.01, 2.09) | 0.21 (0.05, 0.38) | -3.41 (-5.71, -1.12) |
|  | *P* for trend | 0.035 | 0.011 | 0.047 | 0.006 | 0.002 | 0.006 |
| LAP | Quartile 1 | Ref. | Ref. | Ref. | Ref. | Ref. | Ref. |
|  | Quartile 2 | 1.25 (0.86, 1.83) | 0.13 (-0.04, 0.30) | -1.76 (-3.99, 0.47) | 1.17 (0.79, 1.75) | 0.02 (-0.15, 0.16) | -1.55 (-3.75, 0.66) |
|  | Quartile 3 | 1.54 (1.05, 2.27) | 0.24 (0.07, 0.42) | -2.28 (-4.64, -0.93) | 1.34 (0.90, 2.00) | -0.02 (-0.18, 0.14) | -4.18 (-6.49, -1.87) |
|  | Quartile 4 | 1.94 (1.28, 2.92) | 0.38 (0.19, 0.58) | -2.68 (-5.27, -0.09) | 2.40 (1.58, 3.64) | 0.39 (0.21, 0.57) | -2.87 (-5.42, -0.32) |
|  | *P* for trend | 0.001 | <0.001 | 0.007 | <0.001 | <0.001 | 0.006 |
| VAI | Quartile 1 | Ref. | Ref. | Ref. | Ref. | Ref. | Ref. |
|  | Quartile 2 | 1.66 (1.15, 2.39) | 0.16 (-0.01, 0.32) | -2.60 (-4.77, -0.43) | 1.14 (0.78, 1.67) | 0.03 (-0.12, 0.18) | -0.97 (-3.13, 1.20) |
|  | Quartile 3 | 1.44 (0.99, 2.08) | 0.22 (0.05, 0.38) | -2.02 (-5.22, -0.82) | 1.35 (0.93, 1.96) | 0.11 (-0.04, 0.27) | -3.49 (-5.69, -1.29) |
|  | Quartile 4 | 2.04 (1.41, 3.00) | 0.35 (0.18, 0.52) | -2.79 (-5.06, -0.52) | 1.86 (1.29, 2.69) | 0.27 (0.12, 0.43) | -1.80 (-4.04, -0.44) |
|  | *P* for trend | 0.001 | <0.001 | 0.002 | <0.001 | <0.001 | 0.029 |
| CVAI | Quartile 1 | Ref. | Ref. | Ref. | Ref. | Ref. | Ref. |
|  | Quartile 2 | 1.38 (0.94, 2.03) | 0.07 (-0.10, 0.24) | -3.78 (-6.02, -1.54) | 1.13 (0.73, 1.75) | 0.05 (-0.12, 0.22) | -0.88 (-3.08, 1.31) |
|  | Quartile 3 | 1.67 (1.10, 2.54) | 0.24 (0.05, 0.44) | -7.29 (-9.79, -4.79) | 1.58 (0.98, 2.56) | 0.13 (-0.08, 0.33) | -2.49 (-4.89, -0.09) |
|  | Quartile 4 | 2.36 (1.43, 3.92) | 0.43 (0.20, 0.67) | -9.62 (-12.70, -6.54) | 2.65 (1.40, 5.01) | 0.50 (0.22, 0.78) | -4.30 (-7.34, -1.25) |
|  | *P* for trend | 0.001 | <0.001 | <0.001 | 0.002 | 0.003 | 0.001 |

Data are expressed as regression coefficients or odds ratios (95%CI). Linear regression analysis was used for the associations of abdominal obesity index with Ln ACR and eGFR respectively. Logistic regression analyses were used for the association of abdominal obesity index with DKD.

^1^The model was adjusted for age, education, duration of diabetes history, current smoking, BMI, HbA1c, LDL and systolic blood pressure.

^2^The model was adjusted for education, duration of diabetes history, current smoking, BMI, HbA1c, LDL and systolic blood pressure.

Ln ACR, log-transformed albumin to creatinine ratio; eGFR, estimated glomerular infiltration rate; DKD, diabetic kidney disease; BMI, body mass index; HbA1c, glycated hemoglobin; NC, neck circumference; VAI, visceral adiposity index; LAP, the lipid accumulation product; WC, waist circumference; CVAI, Chinese visceral adiposity index; WHR, waist-to-hip ratio; LDL, low-density lipoprotein.

Table S4 Associations between the quartiles of the abdominal obesity indices and the prevalence of DR

|  |  | Men | Women |
| --- | --- | --- | --- |
| NC | Quartile 1 | Ref. | Ref. |
|  | Quartile 2 | 1.34 (0.92, 1.96) | 0.98 (0.67, 1.43) |
|  | Quartile 3 | 1.26 (0.81, 1.96) | 0.94 (0.61, 1.46) |
|  | Quartile 4 | 1.38 (0.83, 2.28) | 1.19 (0.73, 1.91) |
|  | *P* for trend | 0.246 | 0.581 |
| WC | Quartile 1 | Ref. | Ref. |
|  | Quartile 2 | 1.15 (0.77, 1.73) | 0.91 (0.60, 1.36) |
|  | Quartile 3 | 0.73 (0.45, 1.20) | 0.96 (0.61, 1.52) |
|  | Quartile 4 | 0.94 (0.52, 1.71) | 1.09 (0.62, 1.93) |
|  | *P* for trend | 0.466 | 0.759 |
| WHR | Quartile 1 | Ref. | Ref. |
|  | Quartile 2 | 0.92 (0.61, 1.38) | 0.79 (0.54, 1.17) |
|  | Quartile 3 | 0.77 (0.50, 1.88) | 0.82 (0.55, 1.22) |
|  | Quartile 4 | 0.86 (0.55, 1.35) | 0.70 (0.46, 1.07) |
|  | *P* for trend | 0.400 | 0.138 |
| LAP | Quartile 1 | Ref. | Ref. |
|  | Quartile 2 | 0.90 (0.59, 1.36) | 0.95 (0.63, 1.42) |
|  | Quartile 3 | 0.85 (0.55, 1.31) | 1.25 (0.80, 1.93) |
|  | Quartile 4 | 0.72 (0.44, 1.17) | 1.55 (0.89, 2.69) |
|  | *P* for trend | 0.901 | 0.095 |
| VAI | Quartile 1 | Ref. | Ref. |
|  | Quartile 2 | 1.06 (0.71, 1.58) | 0.86 (0.55, 1.33) |
|  | Quartile 3 | 1.10 (0.73, 1.64) | 1.08 (0.66, 1.78) |
|  | Quartile 4 | 0.74 (0.48, 1.15) | 0.96 (0.49, 1.89) |
|  | *P* for trend | 0.922 | 0.849 |
| CVAI | Quartile 1 | Ref. | Ref. |
|  | Quartile 2 | 1.05 (0.70, 1.59) | 0.84 (0.55, 1.27) |
|  | Quartile 3 | 0.67 (0.41, 1.09) | 1.09 (0.69, 1.71) |
|  | Quartile 4 | 0.94 (0.65, 1.71) | 1.11 (0.63, 1.93) |
|  | *P* for trend | 0.450 | 0.533 |

Data are expressed as regression coefficients or odds ratios (95%CI). Logistic regression analyses were used for the association between abdominal obesity indices and the prevalence of DR.

The model was adjusted for age, sex, education, duration of diabetes, current smoking, BMI, HbA1c, LDL and systolic blood pressure.

DR, diabetic retinopathy; BMI, body mass index; HbA1c, glycated hemoglobin; NC, neck circumference; VAI, visceral adiposity index; LAP, the lipid accumulation product; WC, waist circumference; CVAI, Chinese visceral adiposity index; WHR, waist-to-hip ratio; LDL, low-density lipoprotein
